# Efficient construction of Markov state models for stochastic gene regulatory networks by domain decomposition

**DOI:** 10.1186/s12859-025-06174-5

**Published:** 2025-06-02

**Authors:** Maryam Yousefian, Anna-Simone Frank, Marcus Weber, Susanna Röblitz

**Affiliations:** 1 Computational Biology Unit (CBU), Department of Informatics, University of Bergen,, Thormøhlens Gate 55, 5008 Bergen, Norway; 2 Zuse Institute Berlin (ZIB), Takustr. 7, 14195 Berlin, Germany

**Keywords:** Gene regulatory networks, Stochastic phenotype switching, Chemical master equation, Markov state modelling, Robust Perron cluster analysis, Voronoi tessellation

## Abstract

**Background:**

The dynamics of many gene regulatory networks (GRNs) is characterized by the occurrence of metastable phenotypes and stochastic phenotype switches. The chemical master equation (CME) is the most accurate description to model such stochastic dynamics, whereby the long-time dynamics of the system is encoded in the spectral properties of the CME operator. Markov State Models (MSMs) provide a general framework for analyzing and visualizing stochastic multistability and state transitions based on these spectral properties. Until now, however, this approach is either limited to low-dimensional systems or requires the use of high-performance computing facilities, thus limiting its usability.

**Results:**

We present a domain decomposition approach (DDA) that approximates the CME by a stochastic rate matrix on a discretized state space and projects the multistable dynamics to a lower dimensional MSM. To approximate the CME, we decompose the state space via a Voronoi tessellation and estimate transition probabilities by using adaptive sampling strategies. We apply the robust Perron cluster analysis (PCCA+) to construct the final MSM. Measures for uncertainty quantification are incorporated. As a proof of concept, we run the algorithm on a single PC and apply it to two GRN models, one for the genetic toggle switch and one describing macrophage polarization. By comparing the results with reference solutions, we demonstrate that our approach correctly identifies the number and location of metastable phenotypes with adequate accuracy and uncertainty bounds. We show that accuracy mainly depends on the total number of Voronoi cells, whereas uncertainty is determined by the number of sampling points.

**Conclusions:**

A DDA enables the efficient computation of MSMs with quantified uncertainty. Since the algorithm is trivially parallelizable, it can be applied to larger systems, which will inevitably lead to new insights into cell-regulatory dynamics.

## Background

Within a cell, gene expression levels are governed by molecular regulators interacting with each other in gene regulatory networks (GRNs). These networks generate complex dynamical activities like oscillations or multistability that are crucial to the survival, behavior, development, and reproduction of the living cell. Hence, various mathematical models have been developed to study the dynamic behavior of GRNs [1], including Boolean models [2] and ordinary differential equation models [3]. In addition, experimental evidence has been provided that distinct cellular phenotypes correspond to stable attractors in the high-dimensional gene expression state space [4].

It has also been recognized that gene expression is a fundamentally stochastic process [5, 6], where low reactant numbers can lead to significant statistical fluctuations in molecule numbers and reaction rates. These findings have motivated studies of stochastic state-switching in gene networks. For example, stochastic changes in particular patterns of gene expression have been identified with spontaneous phenotype transitions that can diversify otherwise identical cell populations [7]. In terms of mathematical modeling, introducing stochasticity often turns originally irreversible into (weakly) reversible switches. That means particular phenotypes correspond to regions in the state space, which we call *metastable* regions or *metastabilities*, where the dynamics remains for a long time before it rapidly switch to another phenotype. These switchings are *rare events*, i.e., the dynamical system is characterized by a *separation of time scales*.

**Fig. 1:**
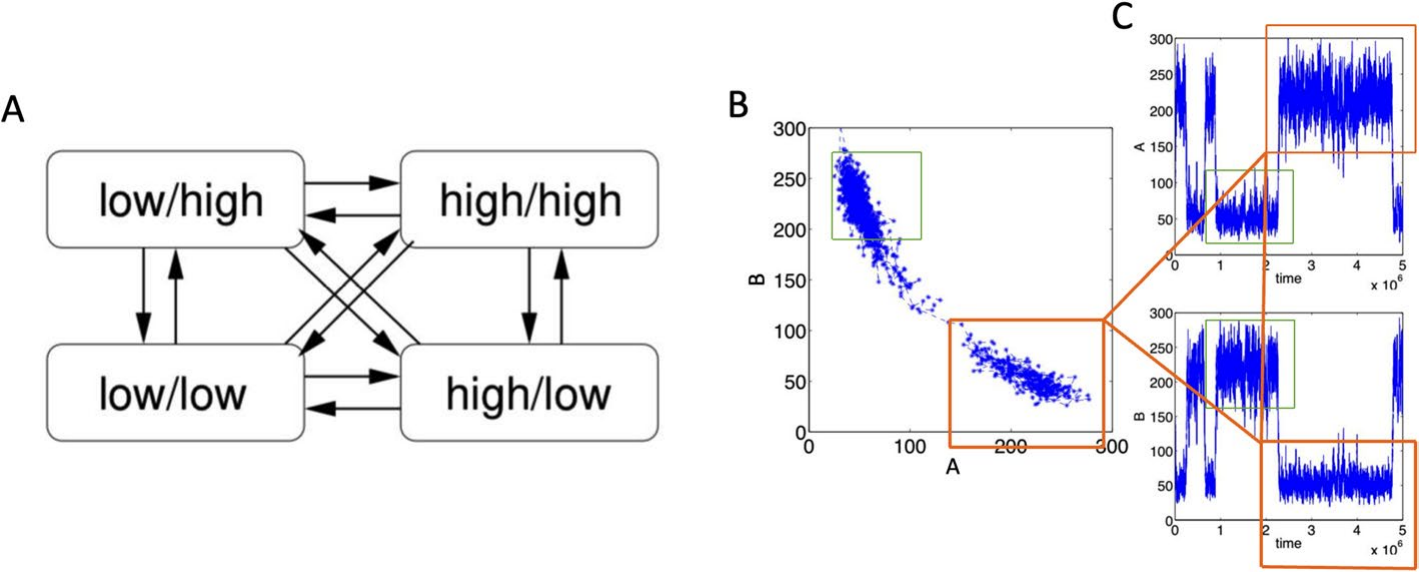
Simulation of phenotype switching. **A** The aim is to identify metastable phenotypic regions in the state space and their corresponding transition patterns. **B** These are regions in the phase space where the dynamics is most likely located. **C** Transitions between these regions are rare events and, therefore, rarely observable in stochastic simulations. For example, the region highlighted by the red rectangles corresponds to a phenotype where *A* is high while *B* is low

Numerous mathematical frameworks have been developed to model and analyze the dynamic behavior of stochastic GRNs dynamics, e.g., stochastic differential equations [8], probabilistic Boolean networks [9], or piecewise-deterministic Markov processes [10]. Even though these models allow for realistic simulations of gene expression data, the most accurate description that accounts for stochasticity due to molecular-level fluctuations and propagates dynamics according to chemical rate laws is given by the Chemical Master Equation (CME) [11, 12]. The CME is a set of ordinary differential equations (ODEs), one ODE for each possible state of the system, whereby a state is a vector containing the discrete entities of the involved species/biomolecules. It describes the time evolution of the probability density in this state space. The dimension of the CME, i.e. the number of possible system states, depends upon the total number of molecules present and the precise form of the chemical reactions and is often very large or even unbounded. Hence, the CME is usually analytically intractable except for some simplified model systems. Nevertheless, trajectories can be generated by the Stochastic Simulation Algorithm (SSA) [13]. For multistable systems, however, the simulation of phenotype switchings would require very long and/or many simulations because exploration of the state space is hindered by rare events. An example involving the dynamics of two species *A* and *B* is shown in Fig. 1. Here, simulations reveal that the system is most likely in one of the two-phase space regions corresponding to A/B being low/high or high/low, with rare transitions between these two phenotypes, whereas the phenotypes low/low and high/high do not occur.

Alternatively, information about phenotypes and their transition patterns can be extracted directly from analyzing the CME operator or, in case of a truncated, finite state space, from the corresponding rate matrix. In particular, the number of metastabilities, i.e. phenotypes, corresponds to the number of eigenvalues close to zero, whereas the location of the phenotypes and their transition patterns can be extracted from the corresponding eigenvectors. This information can subsequently be used to project the high-dimensional dynamical system onto the metastable sets, which results in a low-dimensional approximation of the dynamics on the slow time scales. This reduced representation of a stochastic multistable dynamical system is known as *Markov state model* (MSM) [14]. The MSM contains all information about holding times, transition rates, and transition pathways between the metastable sets (phenotypes). In a proof-of-concept study, Chu et al. [15] found that MSMs provide a general framework for analyzing and visualizing stochastic multistability and state transitions in GRNs. Their study, however, was restricted to two-dimensional systems because constructing the CME rate matrix requires an enumeration of the state space, which becomes tricky in higher dimensions. In a follow-up study from the same group, Tse et al. [16] used a discretization of the state space in terms of Voronoi cells, referred to as micro-states, in combination with a sampling-based approach to obtain a finite-dimensional approximation of the CME operator, from which they derived a MSM. This approach allowed them to build a MSM for systems involving up to eight species. Even though the authors validated their results with SSA simulations, they did not provide any error estimates on the computed quantities. In addition, their approach required the use of high-performance computing (HPC) facilities, which might prevent other researchers from testing this method.

In this paper, we present an alternative and computationally more efficient approach to identifying the dynamics of cellular phenotypes from stochastic GRNs. We suggest combining the domain decomposition approach (DDA) in terms of Voronoi cells with short-time adaptive SSA simulations and statistical error estimation to calculate MSMs with quantified uncertainty. Because this method is trivially parallelizable, our approach paves the way towards constructing MSMs on single workstations with multiple CPU cores, thus making it accessible to a larger research community. We provide a proof-of-concept by applying the approach to two different networks of mutually inhibiting gene pairs with different mechanisms of self-activation. These are frequently occurring motifs in transcriptional regulatory networks to control cell fate decisions.

## Materials and methods

### The chemical master equation (CME)

We consider the dynamics of *D* different species, represented by the state vector ${\textbf{x}}(t)\in \mathbb {N}^D$, under *R* prescribed reactions. Each reaction ${\textbf{x}}(t){\mathop {\longrightarrow }\limits ^{\alpha _r({\textbf{x}})}}{\textbf{x}}(t)+\varvec{\nu }_r$ is described by a transition intensity or reaction propensity $\alpha _r:\mathbb {N}^D\mapsto \mathbb {R}_+$ and the stoichiometric vector $\varvec{\nu }_r\in \mathbb {N}^D$. The master equation is then given by^1^

$$\begin{aligned} \partial _t p({\textbf{x}},t)=\sum _{r=1}^R\left[ \alpha _r({\textbf{x}}-\varvec{\nu }_r)p({\textbf{x}}-\varvec{\nu }_r,t)-\alpha _r({\textbf{x}})p({\textbf{x}},t)\right] =:{{\mathcal {M}}}p({\textbf{x}},t), \end{aligned}$$

where $p({\textbf{x}},t)$ denotes the probability for the system to be in state ${\textbf{x}}$ at time *t*. The adjoint operator ${{\mathcal {M}}}^{*}$, which is defined as the operator satisfying

$$\begin{aligned} \sum _{{\textbf{x}}\in \mathbb {N}^D}{{\mathcal {M}}}p({\textbf{x}})q({\textbf{x}})=\sum _{{\textbf{x}}\in \mathbb {N}^D}p({\textbf{x}}){{\mathcal {M}}}^{*}q({\textbf{x}}) \end{aligned}$$

for any pair (*p*, *q*) of not necessarily normalized or positive functions defined over $\mathbb {N}^D$, is given by

$$\begin{aligned} {{\mathcal {M}}}^{*}q=\sum _{r=1}^R\alpha _r({\textbf{x}})[q({\textbf{x}}+\varvec{\nu }_r)-q({\textbf{x}})]. \end{aligned}$$

In particular, ${{\mathcal {M}}}^{*}$ applied to a constant function yields zero, ${{\mathcal {M}}}^{*}{{{\textbf {1}}}}=0$.

Since the process described by the CME is a time-homogeneous Markov process, there exists a steady state (or stationary) probability distribution $\pi ({\textbf{x}})$ satisfying ${{\mathcal {M}}}\pi ({\textbf{x}})=0\quad \forall \,{\textbf{x}}\in \mathbb {N}^D$. The peaks in this stationary distribution correspond to the metastable regions that we are looking for. These are the regions in which the system is most likely to be found.

Although the CME is infinite in size, the most probable states are those near the metastable regions, and the stationary distribution tends to zero as ${\textbf{x}}$ tends to infinity. Therefore, it is often reasonable to cut the system off and reduce it to some finite state space $\Omega \subseteq \mathbb {N}^D$, thus turning the CME operator ${{\mathcal {M}}}$ into a rate matrix. If the states ${\textbf{x}}\in \Omega$ are numbered $({\textbf{x}}_1,{\textbf{x}}_2,\ldots ,{\textbf{x}}_N)$ such that the reactions can be written as ${\textbf{x}}_i{\mathop {\longrightarrow }\limits ^{\alpha _r({\textbf{x}}_i)}}{\textbf{x}}_j$, then the master operator can be written as reaction rate matrix $K\in \mathbb {R}^{N\times N}$ with elements

$$\begin{aligned} K_{ji}={\left\{ \begin{array}{ll}-\sum _{r=1}^R\alpha _r({\textbf{x}}_i),& \text {for } i=j \\ \alpha _r({\textbf{x}}_i),& \hbox {for all} j \hbox {such that} {\textbf{x}}_j={\textbf{x}}_i+\varvec{\nu }_r\\ 0,& \text {otherwise}\end{array}\right. } \end{aligned}$$

All off-diagonal elements are non-negative, and the columns sum to zero. In matrix notation, the CME reads ${\textbf{p}}'(t)=K{\textbf{p}}(t)$, where ${\textbf{p}}(t)=[p({\textbf{x}}_1,t),p({\textbf{x}}_2,t),\ldots ]^{\top }$. The stationary distribution $\varvec{\pi }=[\pi ({\textbf{x}}_1),\pi ({\textbf{x}}_2),\ldots ]^{\top }$ satisfies $K\varvec{\pi }=0$. However, for algorithmic reasons, it might be easier to work with transition probabilities instead of transition rates.

### From transition rates to transition probabilities

By the Hille-Yosida theorem, ${{\mathcal {M}}}$ is the infinitesimal generator of a strongly continuous semigroup ${{\mathcal {T}}}^{(\tau )}$, and consequently the CME admits a unique continuous solution in the form $p({\textbf{x}},t+\tau )={{\mathcal {T}}}^{(\tau )}p({\textbf{x}},t).$ The corresponding matrix will be denoted by $T(\tau )$. $T(\tau )$ is a row stochastic transition probability matrix. For small time step $\tau$, its entries are given by

$$\begin{aligned} T(\tau )_{ij}=\mathbb {P}[X(t+\tau )={\textbf{x}}_i+\varvec{\nu }_r={\textbf{x}}_j|X(t)={\textbf{x}}_i]=\alpha _r({\textbf{x}}_i)\tau +o(\tau ^2), \end{aligned}$$

but exact analytical expressions are not available. For finite state space, $T^{(\tau )}$ can be computed explicitly as the matrix exponential, $T(\tau )=\exp (\tau K^T)$.

$T(\tau )$ can be used to compute the probability density at multiples of the lag-time $\tau$ via the Chapman–Kolmogorov equation,

$$\begin{aligned} {\textbf{p}}^T(t+k\tau )={\textbf{p}}^T(t)T^k(\tau ). \end{aligned}$$

Moreover, $T(\tau )$ possesses the same eigenvectors as *K*. In particular, the stationary density satisfies $\varvec{\pi }^{\top }T(\tau )=\varvec{\pi }^{\top }$.

### Partitioning into metastable regions

The CME satisfies conservation of mass, i.e. $\sum _{{\textbf{x}}\in \mathbb {N}^D}{{\mathcal {M}}}p({\textbf{x}},t)=0$. In a metastable dynamical system, this mass conservation still applies approximately to the metastable regions. In other words, the probability flux in and out of a metastable set is nearly zero. We denote the number of metastable regions by $n_c$. The specific case $n_c=1$ is not interesting, as it corresponds to processes that are not multistable. In cases where $n_c>1$, we call a partition of unity $\{C_k({\textbf{x}})\}_{k=1}^{n_c}$ of the state space $\mathbb {N}^D$ with $C_k:\mathbb {N}^D\rightarrow [0,1],\,\sum _kC_k({\textbf{x}})=\mathsf {1\!\!\!\;I}_{\mathbb {N}^D}\,\forall \,{\textbf{x}}\in \mathbb {N}^D$ a *metastable function partitioning* if for any probability distribution $p({\textbf{x}},t)$ and all functions $C_k({\textbf{x}})$:

$$\begin{aligned} \sum _{{\textbf{x}}\in \mathbb {N}^D}C_k({\textbf{x}}){{\mathcal {M}}}p({\textbf{x}},t)\approx 0, \quad k=1,\ldots ,n_c. \end{aligned}$$

This definition can be restated in terms of the adjoint ${{\mathcal {M}}}^{*}$ as ${{\mathcal {M}}}^{*}C_k({\textbf{x}})\approx 0$. One possibility to search for such functions $C_k({\textbf{x}})$ is to reformulate the problem as an eigenvalue problem and identify metastable functions as right eigenfunctions of the adjoint operator ${{\mathcal {M}}}^{*}$ for eigenvalues close to zero, $\displaystyle {{\mathcal {M}}}^{*}C_k({\textbf{x}})=\lambda _k C_k({\textbf{x}}), \lambda _k\approx 0$. Correspondingly, the discrete eigenvalue problem reads

$$\begin{aligned} K^T X=X\Lambda ,\quad \Lambda =\text {diag}(\lambda _1,\ldots ,\lambda _{n_C}). \end{aligned}$$

To summarize, metastability is characterized by the occurrence of a cluster of $n_c>1$ eigenvalues $\lambda _1=0>\lambda _2>\ldots >\lambda _{n_c}$ close to zero. Note that the eigenvalue $\lambda _1=0$ is equivalent to the Perron root $\lambda _1=1$ (also called the leading eigenvalue or dominant eigenvalue) of the corresponding $T(\tau )$. Due to this clustering of eigenvalues, the individual eigenvectors are not well-conditioned, but the subspace spanned by all leading eigenvectors is well-conditioned, and the information about the localization of the metastable regions can be extracted from this subspace via the robust Perron cluster analysis (PCCA+) [17].

### Phenotype identification using PCCA+

The aim of PCCA+ is to find a reversible matrix *A* that linearly transforms the eigenvectors *X* into *membership vectors* $\chi =X A$ such that $\chi$ forms a positive partition of unity (i.e. $\chi _j(i)\ge 0 \quad \forall i\in \{1,\ldots ,N\},\,j\in \{1,\ldots ,n_C\}$ and $\sum _{j=1}^{n_c}\chi _j(i)=1\quad \forall i\in \{1,\ldots ,N\}$). The membership vectors $[\chi _1,\ldots ,\chi _{n_c}]$ form a fuzzy clustering of the state space in that they assign to any state the probabilities for belonging to any of the $n_c$ clusters, which represent the metastable regions. In general, *A* is not unique, but PCCA+ finds an optimal transformation such that the entries of the membership vectors are as close as possible to either 1 or 0. It maximizes the so-called crispness of these vectors [17]. The membership vectors not only decode the information about the localization of metastable regions but they can also be used to construct a coarse-grained representation of the GRN dynamics. This is done by projecting the dynamics onto the metastable regions by

$$\begin{aligned} K_c^T=({\chi ^T}\cdot \textrm{diag}(\pi )\cdot \chi )^{-1}\cdot {\chi ^T}\cdot \textrm{diag}(\pi )\cdot K^T \cdot \chi = A^{-1} \cdot \textrm{diag}(\lambda ) \cdot A, \end{aligned}$$

where $\lambda =(\lambda _1,...,\lambda _{n_C})$. The statistical weights $w_k$ of the clusters can be computed by $w_k:=\chi _k^T\pi , k=1,\ldots ,n_c.$ Similarly, the normalized partial densities of the clusters are given by $\pi _k:= \text {diag}(\pi )\cdot \chi _k/w_k.$ While the coarse-grained transition rates might be difficult to interpret, the corresponding coarse-grained transition probabilities for a time interval of length $\tau$ can be obtained via the matrix exponential

$$\begin{aligned} T_c(\tau )=\exp (\tau \cdot K_c^T). \end{aligned}$$

This reduced representation of the dynamics as $T(\tau )$ of a low-dimensional Markov chain is referred to as MSM. The coarse-grained matrices preserve the dominant eigenvalues and, hence, the time scale of the slow processes. Hence, propagation of any projected density vector $\chi ^T{\textbf{p}}$ commutes with the projection of the propagated density $K{\textbf{p}}$,

$$\begin{aligned} K_c\chi ^T{\textbf{p}}=\chi ^TK{\textbf{p}}. \end{aligned}$$

The bottleneck of this approach is the size of the matrix *K* (or $T(\tau )$, respectively). The matrix size increases exponentially with the number of species. Even if truncated to a reasonable finite state space size, and even though *K* is generally sparse, the dimension might still be too large for algorithms based on matrix multiplications, like the one for computing the eigenvalues and eigenvectors. One solution is to decompose the state space $\mathbb {N}^D$ into a finite number of relatively small states, which corresponds to a Galerkin discretization of the CME operator.

### Discretization

Given a partition of unity $\{\psi _i({\textbf{x}})\}_{i=1}^N$, of $\mathbb {N}^D$, the metastable functions $C_k({\textbf{x}})$ can be approximated by

$$\begin{aligned} C_k({\textbf{x}})\approx C_k^N({\textbf{x}})=\sum _{i=1}^N\chi _{ik}\psi _i({\textbf{x}}). \end{aligned}$$

The condition $\sum _k\chi _{ik}=1$ is sufficient to ensure that $\{C_k^N({\textbf{x}})\}$ is a partition of unity if $\{\psi _i({\textbf{x}})\}_{i=1}^N$ is one. Hence, the vectors $\chi _k=(\chi _{1k},\ldots ,\chi _{Nk})^T$ can be interpreted as *membership vectors* whereby the entry $\chi _{ik}\in [0,1]$ indicates the membership of function $\psi _i({\textbf{x}})$ to the metastable function $C_k({\textbf{x}})$.

Using $\displaystyle {{\mathcal {M}}}^{*}C_k({\textbf{x}})=\lambda _k C_k({\textbf{x}}), \lambda _k\approx 0$ and the approximated $C_k({\textbf{x}})$, and forming inner products^2^ with test functions $\{\phi _j({\textbf{x}})\}_{i=1}^N$ we obtain the matrix eigenvalue problem,

$$\begin{aligned} Q\chi _k=\lambda _kS\chi _k, \text { with } Q_{ij}\equiv \langle {{\mathcal {M}}}\phi _i,\psi _j\rangle =\langle \phi _i,{{\mathcal {M}}}^{*}\psi _j\rangle \text { and } S_{ij}\equiv \langle \phi _i,\psi _j\rangle . \end{aligned}$$

Thus, the vectors $\chi _k$ can be computed as right eigenvectors of the matrix pair (*Q*, *S*) corresponding to eigenvalues close to zero. Since the state space is high-dimensional, we must use meshless ansatz functions. One possibility are Voronoi cells. In this case, however, the evaluation of

$$\begin{aligned} Q_{ij}\equiv \langle \phi _i,{{\mathcal {M}}}^{*}\psi _j\rangle =\sum _{{\textbf{x}}\in \mathbb {N}^D} \sum _{r=1}^R\alpha _r({\textbf{x}})[\psi _j({\textbf{x}}+\varvec{\nu }_r)-\psi _j({\textbf{x}})]\phi _i({\textbf{x}}) \end{aligned}$$

would require the detection of all states ${\textbf{x}}$ at the boundary of $\psi _j$ from where the support of $\psi _j$ can be left within one reaction step, which is very difficult or even impossible on unstructured Voronoi tesselations in high dimensions. Another possibility would be to use radial basis functions, but this can deteriorate the condition number of the generalized eigenvalue problem [18]. Alternatively, we can work with transition probabilities. A Galerkin discretization of ${\mathcal {T}}^{(\tau )}$ in terms of Voronoi cells $\{\psi _i({\textbf{x}})\}_{i=1}^N$ and test functions $\phi _i({\textbf{x}})=\psi _i({\textbf{x}})\pi ({\textbf{x}})\equiv \pi _i({\textbf{x}})$ results in the discrete eigenvalue problem $P\chi _k=\lambda _k\chi _k$ with

$$\begin{aligned} P^{(\tau )}_{ij}\equiv \frac{\langle \phi _i,({{\mathcal {T}}}^{(\tau )})^{*}\phi _j\rangle _{\pi }}{\langle \phi _i,\phi _i\rangle _{\pi }}=\frac{\sum _{{\textbf{x}}\in \Omega _i}\sum _{{\textbf{y}}\in \Omega _j}\pi ({\textbf{x}})T^{(\tau )}({\textbf{x}},{\textbf{y}})}{w_i},\quad i,j=1,\ldots ,N. \end{aligned}$$

If we have sampled points $\{{\textbf{x}}_k\}_{k=1}^K$ according to the partial stationary density $\pi _i({\textbf{x}})$, then $P_{ij}$ can be approximated by importance sampling as

$$\begin{aligned} P^{(\tau )}_{ij}\approx \frac{1}{K}\sum _{k=1}^K\sum _{{\textbf{y}}\in \Omega _j}T^{(\tau )}({\textbf{x}}_k,{\textbf{y}}). \end{aligned}$$

Even though we do not know the stationary density point-wise, we can still sample it by running stochastic simulations with SSA. Hence, we present the following algorithm for constructing a MSM.

### Algorithmic approach

**Fig. 2:**
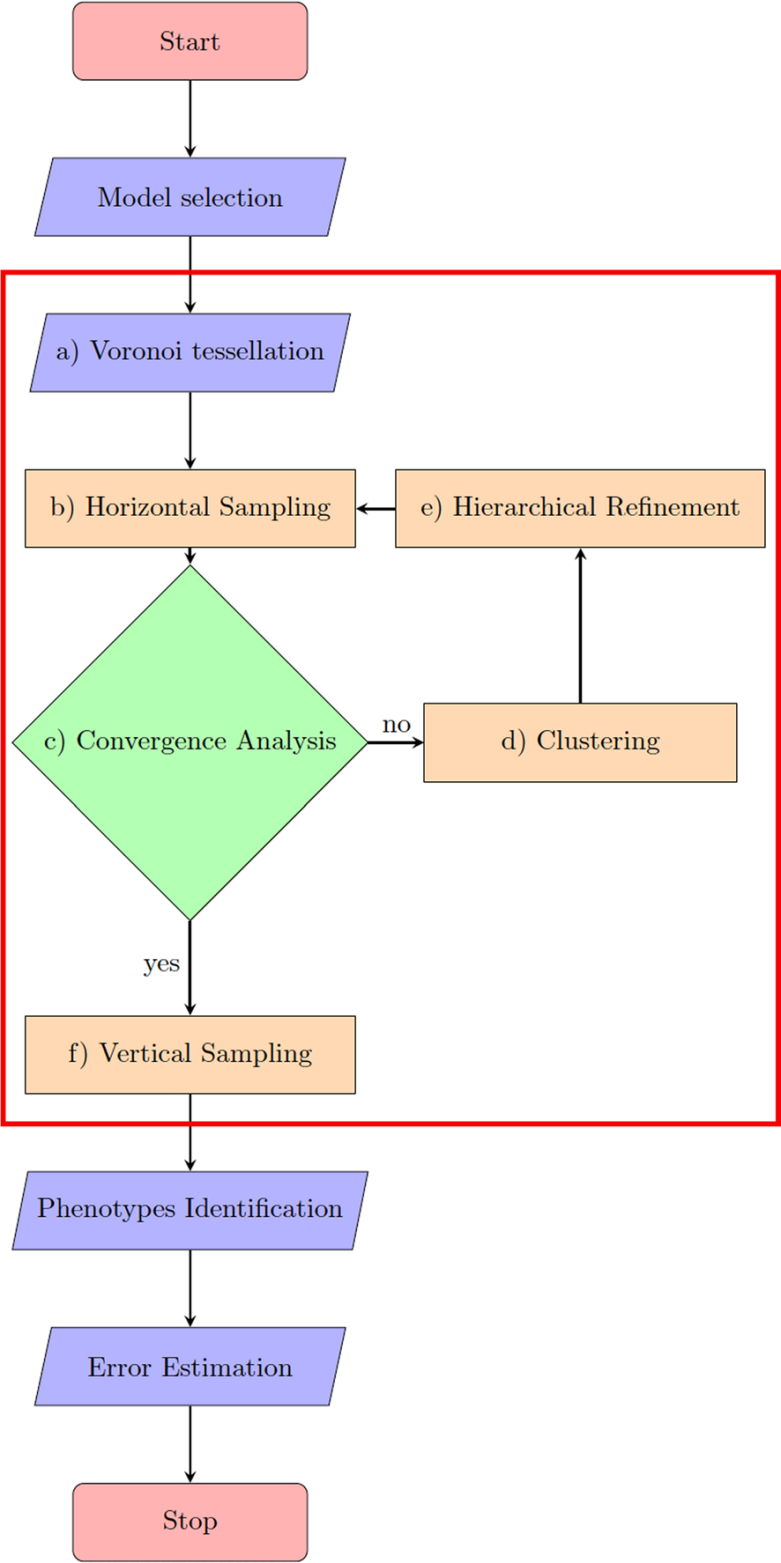
Algorithmic workflow for the current study, starting with the selection of the network to be analyzed within the framework: **a** The state space is decomposed into a finite number of cells represented by their center nodes. **b** The stationary density is sampled within each cell (horizontal sampling). **c** convergence towards the stationary density is checked. If convergence occurs, **f** points are propagated forward over time (vertical sampling). Otherwise, **d**, **e** the horizontal sampling points are clustered to find the location of two new center nodes, and the cell is refined hierarchically. Horizontal sampling and refinement are repeated for the refined cells until convergence is reached. The algorithmic steps within the red box constitute the proposed DDA. Afterward, the PCCA+ algorithm is used to identify metastable regions (phenotypes), and the uncertainty in the model output is estimated

Figure 2 illustrates the main steps of the proposed algorithm, which we will elaborate on in the subsequent subsections a)-f). The algorithmic parameters are explained in Tab. 1.

#### Voronoi tessellation

Given the potentially high-dimensional nature of the state space, we employ meshless methods, particularly Voronoi tessellations. A Voronoi tessellation with center points $\{ {\textbf{x}}_1, \dots , {\textbf{x}}_N \} \in \mathbb {N}^D$ decomposes the state space ${\mathbb {N}^D}$ into a number of $N_0$ cells. Each cell is then described by its characteristic function ${\phi _i}({\textbf{x}}):\mathbb {N}^D\rightarrow \{0,1\},\,i=1,..., N_0$ with

$$\begin{aligned} {\phi _i({\textbf{x}})} = \left\{ \begin{array}{ll} 1; & \quad \text {if } d({\textbf{x}},{\textbf{x}}_i)= \min _{j=1,...,N_0} d({\textbf{x}},{\textbf{x}}_j) \\ 0; & \quad \text {otherwise} \end{array} \right. \end{aligned}$$

where $d({\textbf{x}},{\textbf{x}}_i)$ is a suitable distance function between ${\textbf{x}}$ and ${\textbf{x}}_i$, in our case the Euclidean metric. Note that this decomposition of the state space does not require an a priori truncation of the state space to some finite set $\Omega$. In the current implementation of our algorithm, the coordinates of the Voronoi cells are chosen as uniform random variables rounded to the nearest integer. In higher dimensions, pseudo-random choices are possible. Using Latin hypercubes can lead to high-discrepancy distributions of Voronoi centers in high dimensions. Alternatively, one can place the centers based on prior knowledge, which can be obtained, for example, by generating trajectories with the fast but inexact tau-leaping algorithm [19].

#### Horizontal sampling

The aim of this step is to sample locally from the stationary distribution within each Voronoi cell. For this purpose, we initiate multiple SSA trajectories in the center point of each Voronoi cell. If a trajectory tries to leave the cell, the next state proposed in the SSA algorithm is rejected, and the trajectory stays in its current state until the Markov process jumps to another state within the cell. For each trajectory, we keep track of the time when it first tries to exit the cell. The average of these times over all trajectories that were initiated within the same cell serves as an estimate for the mean first exit time from this cell. In addition, a comparison of the different sampling chains within one cell is used to check for convergence towards the local stationary distribution.

#### Convergence analysis

A necessary criterion for checking the convergence of multiple sampling chains towards the same distribution is the Gelman–Rubin convergence indicator [20]. The criterion compares the within-chain-variances with the between-chain-variances of different trajectories and computes a *potential scale reduction factor *(*R*), which should ideally be close to one [20]. We compute *R* after $N_{\text {hor}}$ horizontal sampling points. If it is still above a defined threshold, namely, $1+R_{\text {thr}}$, then the sampling did not converge towards its equilibrium distribution. Thus, the horizontal trajectories are continued with $N_{\text {hor}}$ more sampling points, and *R* is computed again. As soon as *R* falls below the threshold, the horizontal sampling is stopped. Our assumption is that, in this case, the horizontal sampling has reached an equilibrium. If this does not happen within ${\text {rep}_{\max }}\cdot N_{\text {hor}}$ sampling points, then the basis function is marked for refinement because the non-convergence of the sampling indicated that there is a metastability inside the corresponding cell that has to be resolved by subdividing the cell. This is usually the case when the local stationary density has multiple maxima within a cell, e.g. when it is located on the boundary between two metastable regions. All cells marked for refinement are then ordered according to decreasing mean first exit time, and only the top $\text {ref}_{\max }$ cells are finally refined on the next level. The final goal is to refine the state space in such a way that further discretizations of the transition matrix would not change the spectral information (of the dominant eigenvalues and eigenvectors) anymore. This is the case if the cells either have a high exit rate or the sampling is rapidly mixing within the cells. Rapid mixing would lead to rapid convergence according to the Gelman–Rubin criterion [21].

#### Clustering and hierarchical refinement

The aim of this step is to decompose the cells in which the horizontal sampling did not converge into two new cells. To find the centers of the new cells, we apply *k*-*means* clustering to all horizontal sampling points and use the cluster centers as the center points of the new Voronoi cells. We refine the cells in a hierarchical manner. That means, if a cell with characteristic function $\phi _i({\textbf{x}})$ is decomposed into two new cells $\phi _{i1}({\textbf{x}})$ and $\phi _{i2}({\textbf{x}})$ with center points ${\textbf{x}}_{i1}$ and ${\textbf{x}}_{i2}$, then the characteristic function of $\phi _{i1}$ is defined as

$$\begin{aligned}\phi _{i1}({\textbf{x}})=\left\{ \begin{array}{ll} 1; & \quad \text {if } \phi _i({\textbf{x}})=1 \text { and } d({\textbf{x}},{\textbf{x}}_{i1})<d({\textbf{x}},{\textbf{x}}_{i2})\\ 0; & \quad \text {otherwise} \end{array} \right. \end{aligned}$$

and correspondingly for $\phi _{i2}({\textbf{x}})$. For the refined cells, the algorithm proceeds with horizontal sampling as described in step b). To effectively track the hierarchy of cells, we use a table for bookkeeping the indexes of parental nodes and their respective child nodes. The major advantage of hierarchical refinement is that the sampling only needs to be repeated for the refined cells, while the already converged horizontal samplings of the remaining cells are not affected.

#### Vertical sampling

**Fig. 3:**
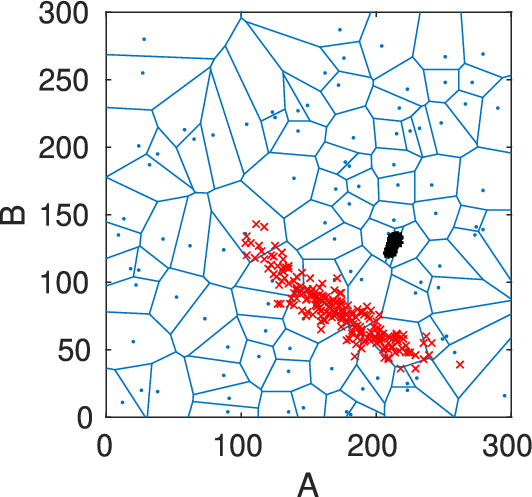
Two-dimensional Voronoi diagram (blue) with 100 cells including the points from horizontal sampling within one specific cell (black) and their end points resulting from the vertical sampling (red)

The aim of this step is to approximate the entries of $T(\tau )$. For this purpose, $N_{\text {vert}}$ points are randomly sampled from the horizontal sampling points within each cell and propagated forward by SSA over a time interval of length $\tau$. For each endpoint located in cell *j*, a transition count of one is added to the *j* entry of a transition count vector, which is finally normalized to obtain a transition probability vector ${\textbf{p}}_i$. This step is illustrated in Fig. 3. The vertical sampling is repeated $N_{\text {r}}$ times for each cell *i*, resulting in a set $D_i=\{{\textbf{p}}_{i1},\ldots ,{\textbf{p}}_{iN_r}\}$ of $N_r$ possible transition probability vectors for row $P^{\tau }(i,:)$. From this set, we compute the maximum likelihood estimate

$$\begin{aligned} P_{\text {mle}}^{(\tau )}(i,:)=\frac{1}{N_r}\sum _{k=1}^{N_r}{\textbf{p}}_{ik}. \end{aligned}$$

This estimate is then used to compute a MSM via PCCA+. Note that $\tau$ should be chosen large enough to avoid some individual cells having very low transition probabilities to other cells, as this might have a negative impact on the accuracy of the estimated MSM. On the other hand, $\tau$ should be as small as possible to save computing time. As a rule of thumb, we use a value of $\tau$ that equals approximately twice the maximum mean first exit time of all cells. As such, $\tau$ could be determined during the simulation run after the horizontal sampling, but we decided to use a fixed value of $\tau$ in each example in order to make the results comparable in case other algorithmic parameters are modified. The major advantage of this sampling-based approach is that we can control the quality of the discretization by checking the convergence behavior of the sampling and controlling the accuracy of the constructed MSM by using re-sampling techniques.

### Error estimation

While the maximum likelihood estimate of $P^{(\tau )}$ provides a point estimate for the mean, it does not convey information regarding uncertainties in these transition probabilities. To address this issue and to quantify the size of the error matrix, it is imperative to estimate the covariance matrix [22]. To choose between the many different possible estimators, we assume that the distribution of probability vectors ${\textbf{p}}_i$ follows a Dirichlet distribution. The Dirichlet distribution, Dir($\varvec{\alpha }$), comprises a group of continuous multivariate probability distributions parameterized by a vector $\varvec{\alpha }$ of positive real numbers. Given a set $D_i=\{{\textbf{p}}_{i1},\ldots ,{\textbf{p}}_{iN_r}\}$ of probability vectors, the classical way of estimating Dirichlet distribution parameters is to maximize the log-likelihood $\log \mathbb {P}(D|\varvec{\alpha })$. This is done by a Newton iteration described in [23] and implemented in the MATLAB toolboxes FASTFIT [24] and LIGHTSPEED [25]. Once an estimate of the Dirichlet parameter vector $\varvec{\alpha }$ is available for each row of $P^{(\tau )}$, we sample a number of $N_{MSM}$ transition probability matrices and compute the corresponding MSM. This allows us to visualize the uncertainty in computed quantities of interest, e.g., eigenvalues or holding probabilities, in terms of boxplots.

### Implementation of illustrative examples

**Fig. 4:**
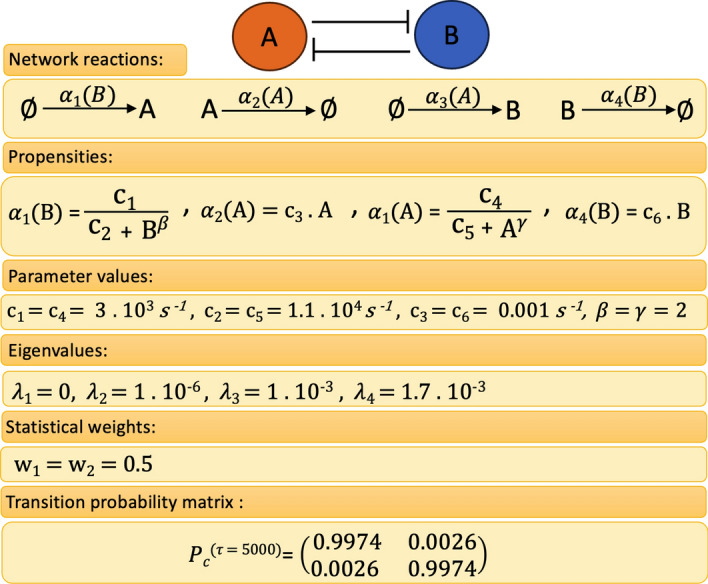
Model A: Representation of the toggle switch network without self-activation including network reactions, propensities, parameter values, eigenvalues, statistical weights, and transition probability matrix with lag time $\tau =5000$

**Fig. 5:**
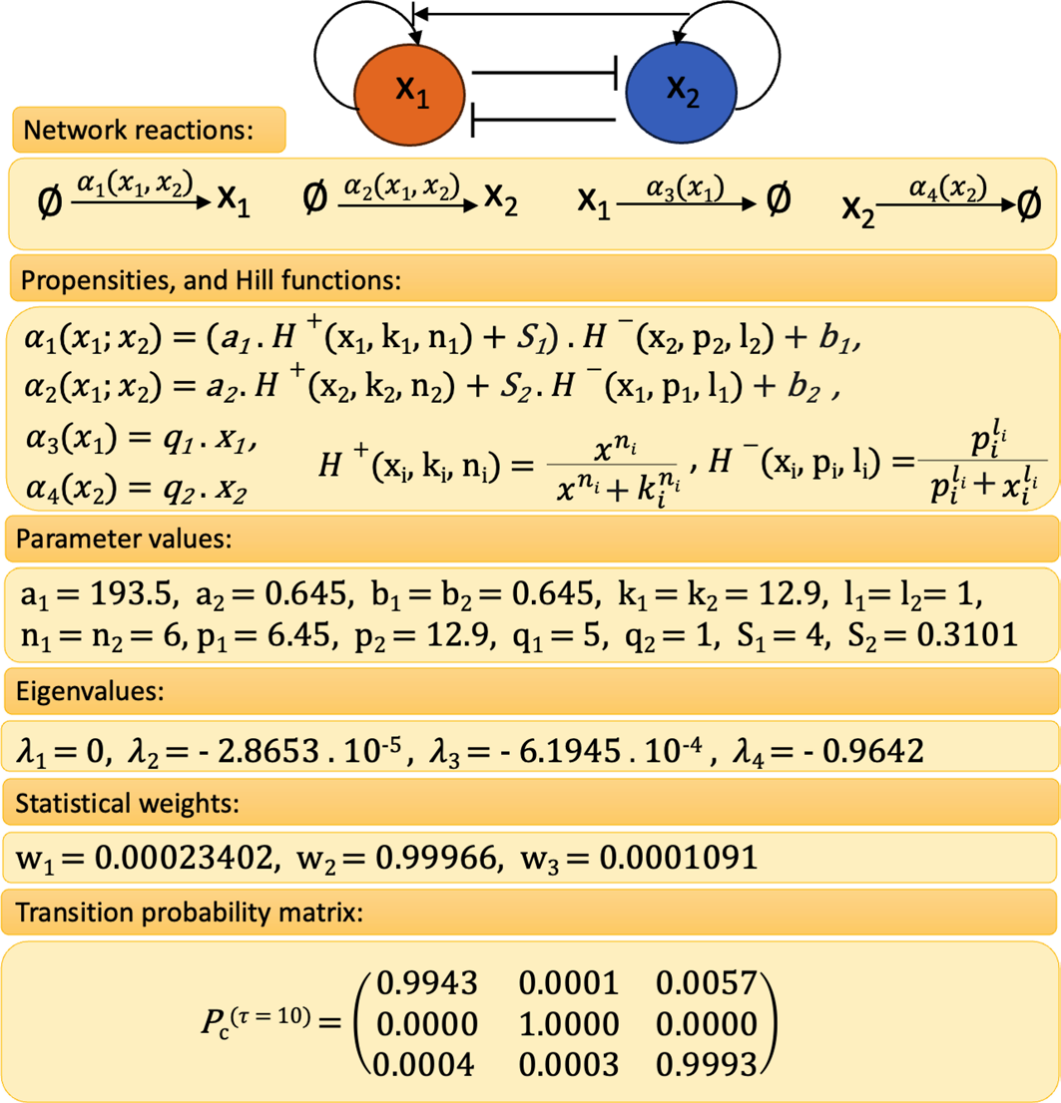
Model B: Representation of the macrophage polarization network including network reactions, propensities, parameter values, eigenvalues, statistical weights, and transition probability matrix with lag time $\tau =10$

All the implementations are done in MATLAB-R2021b, and the codes are available in https://github.com/sroeblitz/MSM2CME. All computations have been performed on a laptop with Intel Core i7-8650U CPU @ 1.90 GHz and 8 MB cache. The random seed in the Matlab code was fixed, and the same for all simulations to ensure the reproducibility of results. We apply our algorithm to two different networks of mutually inhibiting species with different mechanisms of self-activation, as illustrated in Figs. 4 and 5. The example networks were chosen because they nicely illustrate the strengths and weaknesses of our proposed algorithm while at the same time being computationally tractable on a laptop without using parallelization. Table 1 summarizes all algorithmic parameters and their values in the different simulation runs. Parameters that turned out to have a large influence on the accuracy and uncertainty of the solutions were varied throughout different runs. The parameters for horizontal sampling were initially chosen and adapted to obtain convergence towards the local stationary densities for all cells. The parameters for uncertainty quantification were chosen so that an increase in these values did not lead to visible changes in the estimated confidence intervals.

**Table 1:** Algorithmic parameters and their values in different simulation scenarios (A1, A2, A3, and B)

Parameter	Explanation	A1	A2	A3	B
*Model-specific parameters:*					
$N_x,N_y$	Size of the domain $[0,N_x]\times [0,N_y]$ in which the initial Voronoi cells are placed	300	300	300	100
$\tau$	lag time of the $T(\tau )$	5000	5000	5000	10
*Parameters with large influence on accuracy and uncertainty:*					
$N_0$	Initial number of Voronoi cells	100	100	**20**	100
$\text {level}_{\max }$	Maximum hierarchy level	3	3	**5**	3
$N_{\text {vert}}$	Number of vertical sampling points for each cell	**100**	500	500	200
*Parameters for horizontal sampling:*					
$N_{\text {hor}}$	Number of horizontal sampling points until first convergence check	500	500	500	500
$N_{\text {traj}}$	Number of horizontal trajectories within each cell	5	5	5	5
$\text {rep}_{\max }$	Maximum number of horizontal samplings before final convergence check	5	5	5	5
$R_{\text {thr}}$	Threshold $1+R_{\text {thr}}$ for Gelman–Rubin convergence criterion	0.03	0.03	0.03	0.03
$\text {ref}_{\max }$	Maximum number of cells to be refined on one level	10	10	10	10
*Parameters for uncertainty quantification:*					
$N_{r}$	Number of candidate rows for each cell	10	10	10	10
$N_{\text {MSM}}$	Number of sampled transition probability matrices for visualizing uncertainties	20	20	20	20

The values in bold are those that are different from the other scenarios.

## Results

### Model A: toggle switch

The toggle switch is composed of two repressors, further called A and B, and two constitutive promoters. Each promoter is inhibited by the repressor that is transcribed by the opposing promoter. We describe the mutual inhibition of the two genes using a stochastic, highly simplified two-stage model of gene expression based on the deterministic model presented in [26]. This model includes the synthesis and degradation of the two repressors as well as parameter values that preserve the symmetry in the system’s topology.

**Fig. 6:**
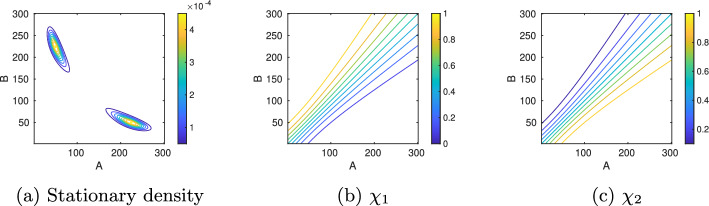
Model A: Reference solution on the truncated state space $\Omega =[0,300]\times [0,300]$ including the stationary density as well as the two cluster membership functions $\chi _{1,2}$

In Fig. 4, the toggle switch and its reactions, propensities, parameter values, eigenvalues, statistical weights, and transition probability matrix with $\tau =5000$ are shown. Spectral analysis of the full CME matrix on the domain $[0,300]\times [0,300]$ reveals a cluster of two eigenvalues close to zero, followed by two eigenvalues further away from zero (Fig. 4). The coarse-grained $P^{(\tau )}$ for $\tau =5000$ serves as a reference solution to which we compare the MSM obtained with the DDA. Application of PCCA+ for $n_c=2$ clusters results in two equally weighted membership functions which separate the state space along the diagonal $A=B$ (Fig. 6b and c).

#### Case A1: discretization and local sampling reveal the metastabilities

**Fig. 7:**
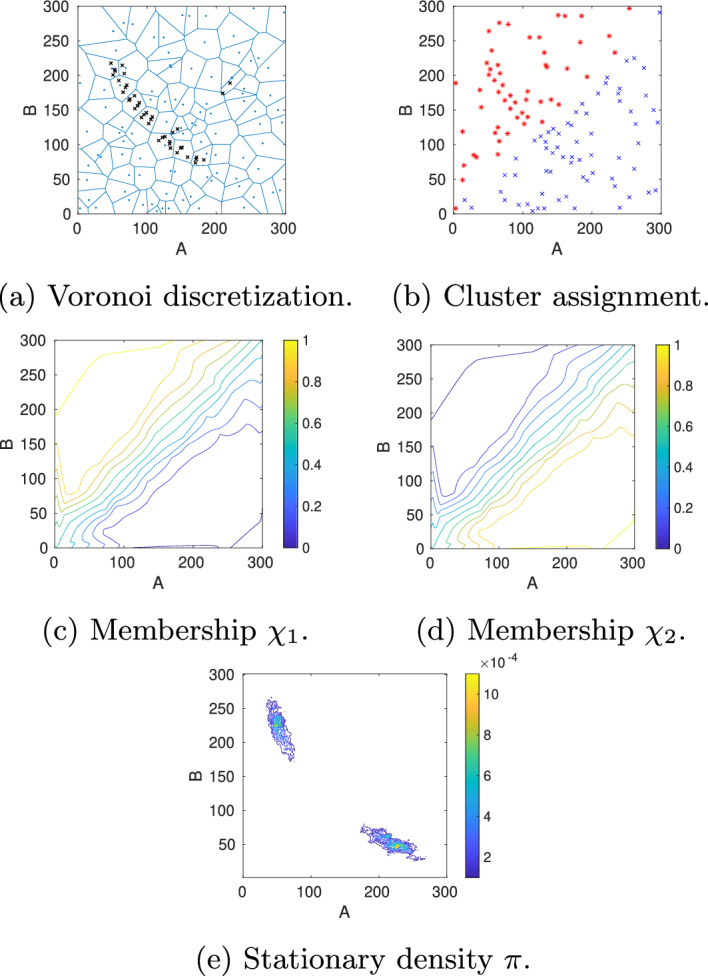
Model A (case A1): **a** For an initial start discretization with $N_0=100$ randomly located Voronoi cells (blue lines), the adaptive sampling and refinement strategy automatically places new nodes (black crosses) along the support of the stationary density as well as in the transition region between the two metastable regions. **b** Based on the approximated transition probability matrix, the individual cells, represented by their center nodes, are assigned to one of the two basins of attraction (blue or red). **c),d)** This assignment is based on the values of the two approximated membership functions $\chi _{1,2}$: Nodes were colored according to the cluster to which they belong with the highest membership. **e)** Stationary density was obtained by re-weighting the locally sampled densities with the stationary weights of the Voronoi cells

To illustrate our approach, we start with an initial discretization into 100 Voronoi cells and initialize 5 trajectories in each cell. The Gelman–Rubin convergence estimator is computed every 500 sampling steps, whereby the maximum length of the horizontal samplings is set to $5\cdot 500$ steps. At most 10 cells are marked for refinement, and the maximum refinement level is 3. Figure 7a shows how the adaptive sampling automatically refines the Voronoi cells placed in the support of the stationary density as well as in the transition region between the two metastable regions, resulting in a total of 120 Voronoi cells on the last level. For the vertical sampling, we propagate from each cell 100 points picked randomly from the horizontal sampling points over a lag time $\tau =5000$. This vertical sampling is repeated 10 times, resulting in each cell in 10 candidate probability vectors from which the parameter vector $\alpha$ of the Dirichlet distribution is estimated for the corresponding row. The overall computing time for this setting is about 25 min, whereby the exact time depends on the selected random seed. We then applied PCCA+ to the maximum likelihood estimate of $T(\tau )$, $P_{\text {mle}}^{(\tau )}$, to construct an estimate of the MSM. This analysis reveals two cluster ($\lambda _2=0.9950$) with weights $w_1=0.48386,\, w_2=0.51614$ and transition probability matrix

$$\begin{aligned}P_c^{(5000)}= \begin{pmatrix} 0.9974 & 0.0026\\ 0.0024 & 0.9976\\ \end{pmatrix}, \end{aligned}$$

which approximates the reference MSM quite well. The two membership functions $\chi _{1,2}$ are plotted in Fig. 7c and d). Moreover, the location of the stationary density $\pi$ in Fig. 7e agrees well with the location of the density in the reference solution (Fig. 6a). However, the question remains whether the inaccuracies in comparison to the reference MSM are due to the discretization error, i.e. the error made in the approximation of membership functions by Voronoi cells, or due to a sampling error in approximating the entries of the transition probability matrix $P^{(\tau )}$. To find an answer to this question, we repeat the numerical experiment with a different number of Voronoi cells and a different number of vertical sampling points.

#### Case A2: increasing the number of vertical sampling points reduces the uncertainty

We repeat the numerical experiment from the previous section, but this time we increase the number of vertical sampling points per cell to $N_{\text {vert}}=500$. All remaining algorithmic parameters remain the same. The overall computing time increases to about 2 h. Again, by applying PCCA+ to the maximum likelihood estimate $P_{\text {mle}}^{(\tau )}$, we obtain two clusters ($\lambda _2=0.9947$) with weights $w_1=0.49246,\, w_2=0.50754$ and transition probability matrix

$$\begin{aligned}P_c^{(5000)}= \begin{pmatrix} 0.9973 & 0.0027\\ 0.0026 & 0.9974 \end{pmatrix}. \end{aligned}$$

**Fig. 8:**
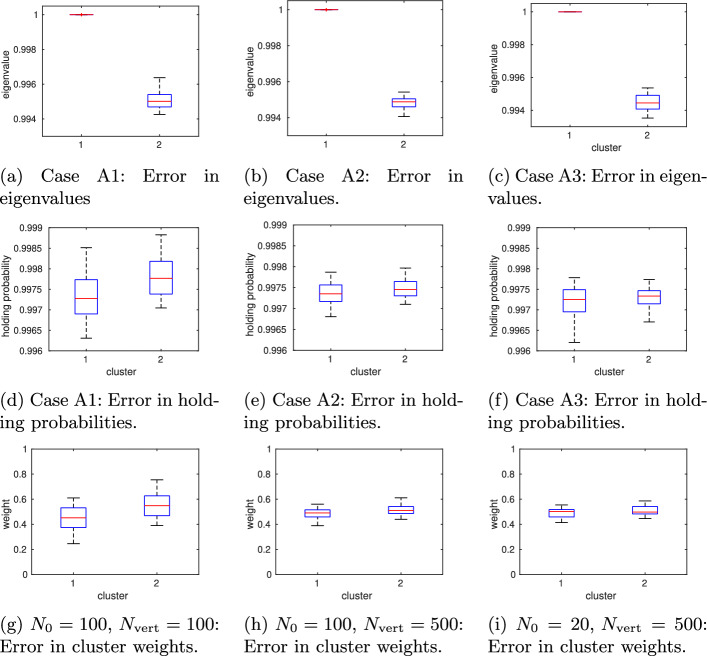
Model A: Increasing the number of vertical sampling points from $N_{\text {vert}}=100$ (left) to $N_{\text {vert}}=500$ (middle) while maintaining the same number of initial Voronoi cells ($N_0=100$) and horizontal sampling points ($N_{\text {vert}}=500$) reduces the uncertainty in the computed quantities of interest. Decreasing the number of initial Voronoi cells, $N_0$, from 100 to 20 (right) does not deteriorate this gain in accuracy

Therefore, the symmetry observed in the reference solution is now better restored. While no difference is visible by eye in the plotted membership functions and partial densities, the decrease in uncertainty becomes visible when multiple MSMs are constructed from sampled transition probability matrices. The boxplots in Fig. 8 (middle column) show that the uncertainty in estimated quantities like the second eigenvalue, holding probabilities $P_{ii}^{(\tau )}$, or cluster weights $w_{1,2}$ significantly decreases when the number of vertical sampling points is increased.

#### Case A3: decreasing the number of Voronoi cells does not deteriorate the discretization error

To examine the influence of the discretization error, we keep the number of vertical sampling points at $N_{\text {vert}}=500$ but decrease the number of initial Voronoi cells to $N_0=20$ while increasing the maximum hierarchy level to $\text {level}_{\max }=5$ to enable sufficient refinement. All remaining algorithmic parameters remain the same. The hierarchical refinement results in a total of $N=60$ Voronoi cells, and the overall computing time is about 1 h. The second eigenvalues of $P_{\text {mle}}^{(\tau )}$ is $\lambda _2=0.9946$. The two clusters have weights $w_1=0.5036,\, w_2=0.4964$ and transition probabilities

$$\begin{aligned}P_c^{(5000)}= \begin{pmatrix} 0.9973 & 0.0027\\ 0.0027 & 0.9973\\ \end{pmatrix}. \end{aligned}$$

**Fig. 9:**
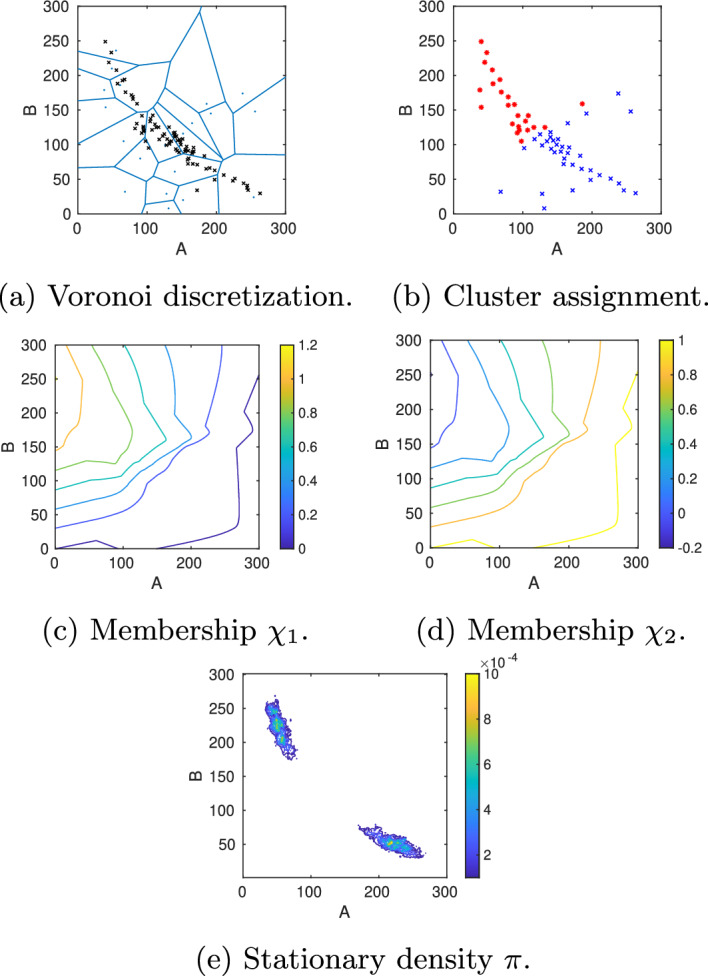
Model A (case A3): **a** For an initial start discretization with $N_0=20$ randomly located Voronoi cells (blue lines), the adaptive sampling and refinement strategy automatically places new nodes (black crosses) along the support of the stationary density as well as in the transition region between the two metastable regions. **b** The individual cells, represented by their center nodes, are assigned to one of the two basins of attraction (blue or red). To simplify visualization, nodes have been colored according to the cluster to which they belong with the highest membership. **c**, **d** This assignment is based on the values of the two approximated membership functions $\chi _{1,2}$: Nodes are colored according to the cluster to which they belong with the highest membership. **e** Stationary density was obtained by re-weighting the locally sampled densities with the stationary weights of the Voronoi cells

The two membership functions $\chi _{1,2}$ are plotted in Figs. 9cand 9d), though not as symmetric as in the reference solution because the second cluster $\chi _2$ (high A, low B) stretches slightly into the region above the diagonal. However, since this mainly happens in the region where the stationary density is low, it does not affect the symmetry of the dynamics , which is also visible in the symmetry of the stationary density in Fig. 9eand 8i).

### Model B: macrophage polarization

**Fig. 10:**
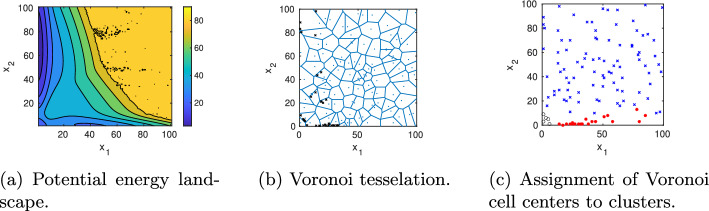
Model B: **a** The potential energy landscape $U(x)=-\log (\pi (x))$ constructed from the full CME clearly shows three different basins of attraction corresponding to $x_1/x_2$ being low/high, low/low or high/low. **b** The adaptive sampling and refinement places new cells at the boundaries of the local stationary densities and in the transition regions. **c** The clustering algorithm assigns each Voronoi cell center to one of the three metastable regions

**Fig. 11:**
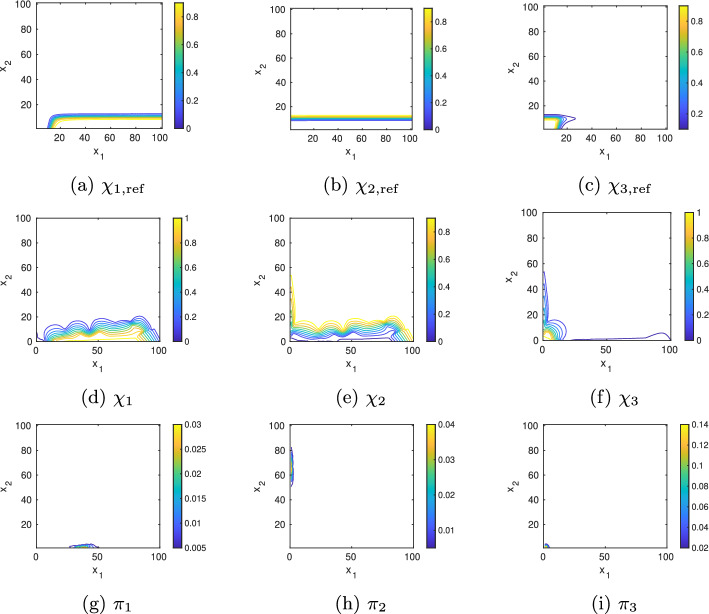
Model B: Contour plot of the corresponding membership functions $\chi _{1,2,3}$ computed from the full CME matrix (**a**–**c**) and their approximation by Voronoi cells (**d**–**f**) as well as the approximated partial stationary densities (**g**–**i**)

The macrophage polarization model presented in [27] consists of four elementary reactions corresponding to synthesis and degradation of STAT1 ($x_1$) and STAT6 ($x_2$), respectively, as well as four stochastic propensity functions presented in Fig. 5. We consider a parameter set from [27] for which the system has three metastable regions corresponding to STAT1/STAT6 being low/high, low/low, or high/low, as visualized by the potential energy landscape $U(x)=-\log (\pi (x))$ in Fig. 10afollowed by a gap to the fourth smallest eigenvalue. The parameter values, eigenvalues, and statistical weights are listed in Fig. 5. Application of PCCA+ to the corresponding eigenvectors results in a decomposition of the state space in terms of three membership functions $\chi _{1,2,3}$ as illustrated in Figs. 11a–c with statistical weights and transition probability matrix shown in Fig. 5. All three clusters are clearly metastable, but most of the statistical weight is on cluster 2 (low/high), which also has the largest basin of attraction. This makes it very difficult to detect the other two attractors in SSA simulations that are initiated outside their basins of attractions. In contrast, our domain decomposition approach with $N_0=100$ cells and $N_{\text {vert}}=200$ sampling points clearly identifies the three metastable regions in terms of their corresponding membership functions (Figs. 10 and 11). During the horizontal sampling, the algorithm particularly refines cells located at the boundary of cluster 1 as well as cells close to the support of the stationary density $\pi (x)$, resulting in a total of 117 cells on the last refinement level 3 (Fig. 10b). The computing time for this example was about 35 min.

The maximum likelihood estimate $P_{mle}^{(\tau )}$ has a cluster of three eigenvalues close to one ($\lambda _1=1,\,\lambda _2=0.9993,\,\lambda _3=0.9944$), and PCCA+ returns three clusters with weights $w_1=0.0038,\,w_2= 0.0043,\, w_3=0.9918$ and transition probability matrix

$$\begin{aligned}P_c^{(10)}= \begin{pmatrix} 0.9945 & -0.0009 & 0.0064\\ -0.0006 & 0.9992 & 0.0014\\ 0.0000 & 0.0000 & 1.0000\\ \end{pmatrix} \end{aligned}$$

**Fig. 12:**
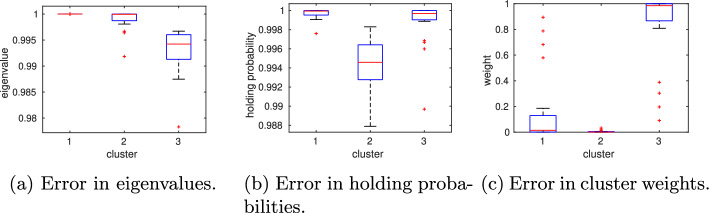
Model B: The eigenvalues **a** and holding probabilities **b** are approximated with reasonably small uncertainty, while the cluster weights **c**cannot be inferred with sufficiently small uncertainty

**Fig. 13:**
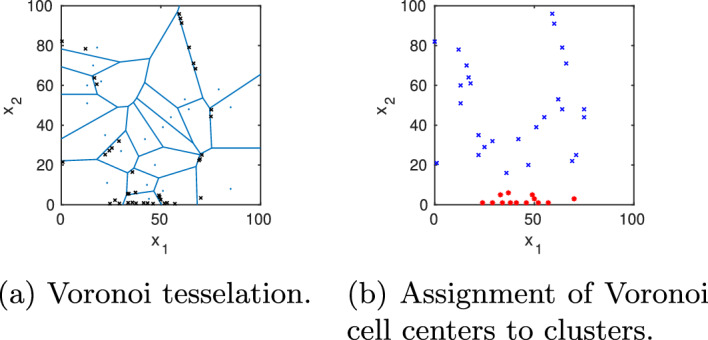
Model B: If none of the cell centers is placed in the lower left corner (**a**), i.e., in the basin of attraction for the cluster where both $x_1$ and $x_2$ are small, then this metastable region is not detected by the algorithm (**b**)

Note that negative entries in this matrix can occur due to the membership vectors not being perfectly zero or one [17]. However, these negative entries are small. The major difference to the reference solution is in the statistical weights. Since the exit probability from cluster 3 is approximated as being almost zero, its statistical weight is close to one, in contrast to the reference solution, where cluster 2 gets the highest statistical weight. When sampling multiple MSMs, cluster 3 is assigned the lowest statistical weight in almost all scenarios, in some cases cluster 1 has the highest weight, whereas cluster 2 never dominates (Fig. 12c). Increasing the number of vertical sampling points to $N_{\text {vert}}=500$ was insufficient to improve the weights’ approximation. Note that if the number of initial Voronoi cells is decreased, e.g. to $N_0=20$, it might even happen that none of the initial Voronoi centers is placed in the basin of attraction of the low/low cluster, in which case it could not be detected by the algorithm (Fig. 13).

### Comparison with SSA

**Fig. 14:**
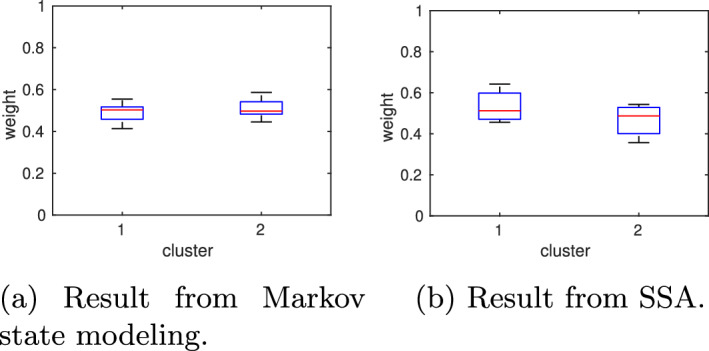
Comparison of cluster weights obtained with the Markov state modeling approach (**a**) and SSA (**b**)

We compared the results from our algorithm applied to models A and B with the results obtained from running long-time simulations with Gillespie’s stochastic simulation algorithm [13]. For model A, case A3 generated the lowest number of sampling points compared to cases A1 and A2 while achieving a similar accuracy as A1 and uncertainty as A2. In total, case A3 used 60 Voronoi cells on the deepest hierarchy level and $N_\text {vert}=500$ initial points for vertical sampling within each cell, i.e. a total of $60\cdot 500$ trajectories of length $\tau =5000$ were run (the number of horizontal sampling points is negligible as it was three orders of magnitude lower). This was repeated $N_r=10$ times to quantify the uncertainty in the results. To compare it with SSA using the same amount of sampling points, we generated 10 SSA trajectories, each starting in the point [1, 1] and running until a final time $t_f=60\cdot 500\cdot 5000=15\cdot 10^7$. For each of these 10 trajectories, we computed the statistical weights of the two metastable regions by comparing the number of sampling points above the diagonal $A=B$ to the number of points below the diagonal, whereby points on the diagonal were assigned 50/50 to each of the two clusters. Figure 14 shows that the uncertainty in the cluster weights obtained from SSA is larger than in the results obtained from the MSM approach despite a similar number of sampling points used. The main reason is that transitions between the two metastable regions are rare events, but they need to happen often enough in a single SSA trajectory to obtain the right statistical weights. Hence, the SSA trajectory would need longer to obtain a better result.

The results for model B in the previous subsection were based on 117 Voronoi cells and $N_\text {vert}=200$ vertical sampling trajectories, each of length $\tau =10$. To compare the results with SSA, we generated 10 trajectories, each with final time $t_f=117\cdot 200\cdot 10=2.34\cdot 10^5$. We initiated these trajectories at 10 randomly selected points in the domain $[0,100]\times [0,100]$. However, only one of these trajectories, namely one initiated in the first metastable region, visited all three metastable regions (with weights $w_1=0.0011,\,w_2=0.9624,\,w_3=0.0365$.^3^ All other trajectories already started in or directly went to the second metastable region and stayed there until the end of the simulation time. This illustrates that transition probabilities or rates between metastable regions in model B are difficult to obtain with either of the two methods (domain decomposition or SSA) and that alternative approaches are needed for this model.

To estimate holding probabilities from an SSA trajectory, one could estimate the transition rates between the metastable regions and obtain the probabilities from the matrix exponential of the rate matrix. Transition rates in model A can be estimated, for example, by counting the number of transitions between the sets $A>B$ and $A<B$. However, this would yield higher rates and thus lower holding probabilities than the Markov state modeling approach due to the different definitions of the metastable regions (soft membership functions $\chi _{1,2}$ versus indicator functions of subsets). In principle, one could also build a MSM based on the long-time SSA trajectories to compute these membership functions. This approach would again require some discretization of the phase space but would not benefit from adaptive sampling and refinement as our domain decomposition approach.

## Discussion

Our algorithm provides a tool for the theoretical examination of stochastic state-switching within gene networks. The results show that the proposed domain decomposition approach is capable of discovering all metastable regions in stochastic gene regulatory networks with multiple attractors and approximating the membership functions with good accuracy. A metastable region is found as long as at least one initial center node is located in its basin of attraction. If the basins of attraction are large enough, this usually happens even with randomly placed initial cells. However, metastable regions with a very small basin of attraction might be missed in this approach, as we have illustrated for model B.

The algorithm also succeeds in approximating the partial stationary densities within the identified metastable regions as well as their global weights, as illustrated for model A. However, the approximation of the global statistical weights fails if the clusters are dynamically well separated. This is not surprising as the stationary density of an almost decoupled Markov chain is known to be highly ill-conditioned [28, 29]. However, once the partial stationary densities have been located, there exist alternative methods to correctly infer transition rates between them, for example transition path sampling [30, 31]. Another solution would be to increase the lag time $\tau$ in our algorithm, which would also increase the overall computing time. Typically, horizontal sampling is fast, whereas vertical sampling takes most of the computing time. This time scales linearly with both the number of vertical sampling points, $N_{\text {vert}}$, the number of candidate rows, $N_r$, and the lag time $\tau$.

In general, the accuracy of the approximated MSM depends mainly on the number of Voronoi cells, whereas the uncertainty is largely influenced by the number of vertical sampling points. Thus, if the main point of interest is to explore the number and location of metastable phenotypes, the algorithm can be run quickly with a low number of vertical sampling points. Since the CME is only a model itself, the modeling error might dominate the approximation error of the MSM. Still, understanding the characteristics of metastable regions and the barriers separating them provides critical insights into the stability and transition dynamics of gene expression profiles. In particular, elucidating the critical points where stochastic state-switching events are more likely to occur, further deepens our comprehension of the regulatory mechanisms governing gene expression variability.

Although we have implemented the algorithm for only two-dimensional systems so far, the use of a meshless discretization makes it easily extendable to higher dimensional systems and other biological applications where multistability is expected to occur.

An alternative approach for deriving a MSM for stochastic phenotype transitions would be to model gene expression as a piecewise-deterministic Markov process (PDMP), for which analytical results can be exploited [32]. This approach also makes use of metastability for reverse engineering of the GRN topology from single-cell RNA sequencing data [33]. A comparison between the CME and PDMP modeling approach in terms of accuracy and efficiency was out of the scope for this work here, but will be an interesting avenue to explore in future.

Our main future work will be to parallelize the domain decomposition algorithm. Since both the horizontal as well as the vertical samplings are independent between different cells, the proposed algorithm is trivially parallelizable in that the samplings for different cells can be run in parallel. In addition, different horizontal sampling chains within one cell could run in parallel as well. This possibility for parallelization paves the way towards the application to higher dimensional problems, though high-performance computing facilities might be needed for systems with a large accessible state space.

## Conclusion

We have proposed an algorithm for the computation of MSMs, which is based on local adaptive samplings and the statistical assembly of these trajectories into a global transition probability matrix. In our approach, many local short-term simulations can be combined to figure out the long-term global behavior of the system. The method is also adaptive such that computational costs are saved, and sampling data is only generated where it is “needed”. Our algorithm identifies basins of attraction of metastable regions as well as the local stationary densities with moderate computational effort. It also succeeds in approximating their statistical weights and transition probabilities as long as the lag time of the MSM is chosen large enough to capture sufficiently many transitions between the metastable regions. Our algorithm is trivially parallelizable, which paves the way for its application to larger systems.

## Data Availability

The datasets generated and/or analyzed during the current study as well as the code for generating the figures are available in the GitHub repository https://github.com/sroeblitz/MSM2CME. A release has been uploaded to Zenodo: https://doi.org/10.5281/zenodo.15468657. The two gene regulatory networks that were used as proof of concept are also fully specified in terms of their equations and parameters within the manuscript. The data for the toggle switch model were taken from [26] and are based on a synthetic gene-regulatory network in Escherichia coli. The data for the macrophage model were taken from [27] and relate to experimental findings in diverse macrophage cell lines as summarized in [34] and [27].

## Footnotes

1 We postulate that $\alpha _r({\textbf{x}})=0$ if $\exists i: x_i<0$ or $\exists i:x_i+(\nu _r)_i<0$.

2 We consider inner products in the weighted $l^2$ sequence space, ${{\mathcal {H}}}_{\eta }=\{u:\mathbb {N}^D\rightarrow \mathbb {R}:\sum _{{\textbf{x}}\in \mathbb {N}^D}u({\textbf{x}})^2\eta ({\textbf{x}})<\infty \}$ with $\langle u,v\rangle _{\eta }\equiv \sum _{{\textbf{x}}\in \mathbb {N}^D}u({\textbf{x}})v({\textbf{x}})\eta ({\textbf{x}}).$ In the unweighted sequence space where $\eta ({\textbf{x}})\equiv \mathsf {1\!\!\!\;I}_{\mathbb {N}^D}$, we simply omit $\eta$ in the notation.

3 We defined the metastable regions by the three sets $S_1=\{x | x_1\ge 13\, \& \, x_2\le 10\}$, $S_2=\{x | x_2\ge 11\}$, and $S_3=\{x | x_1\le 12\, \& \, x_2\le 10\}$.
